# Differences in Plasma Extracellular Vesicles of Different Origin in On-Pump Versus Off-Pump Cardiac Surgery

**DOI:** 10.3390/cimb46110779

**Published:** 2024-11-17

**Authors:** Arthur Aquino, Napisat Abutalimova, Yi Ma, Imran Ismail-zade, Vadim Grebennik, Artem Rubinstein, Igor Kudryavtsev, Ekatherina Zaikova, Darina Sambur, Alexander Marichev, Olga Kalinina, Andrey Bautin, Anna Kostareva, Jarle Vaage, Alexey Golovkin

**Affiliations:** 1 Almazov National Medical Research Centre, 197341 St. Petersburg, Russia; akino97@bk.ru (A.A.); abutalimova@inbox.ru (N.A.); mayi29082@yandex.ru (Y.M.); imran-zade@mail.ru (I.I.-z.); grebennik_vk@almazovcentre.ru (V.G.); arrubin6@mail.ru (A.R.); igorek1981@yandex.ru (I.K.); catherine3452@yandex.ru (E.Z.); sambour-darina@mail.ru (D.S.); doc@amarichev.ru (A.M.); olgakalinina@mail.ru (O.K.); abautin@mail.ru (A.B.); anna.kostareva@ki.se (A.K.); 2 Institute of Experimental Medicine, 197022 St. Petersburg, Russia; 3 Oslo University Hospital, University of Oslo, 0372 Oslo, Norway; i.j.vaage@medisin.uio.no

**Keywords:** extracellular vesicles, coronary artery bypass grafting, on-pump heart surgery, off-pump heart surgery

## Abstract

Coronary artery bypass grafting (CABG) using cardiopulmonary bypass (CPB) causes a systemic inflammatory response that can worsen patient outcomes. Off-pump surgery has been associated with a reduced inflammatory response. The precise mechanisms and the role of extracellular vesicles (EVs) in this context are not fully understood. This study aimed to investigate the early immune response, including main T- and B-lymphocyte subsets, cytokine profiles, and plasma EVs, in patients undergoing off-pump (*n* = 18) and on-pump (*n* = 18) CABG. Thirty-six patients undergoing isolated CABG were enrolled in this randomized control study. Pre- and 24 h postoperative blood samples were analyzed for immune cell populations, cytokine levels, and plasma EV phenotyping. Off-pump CABG triggered a milder immune response than on-pump surgery. On-pump surgery led to greater changes in circulating EVs, particularly platelet- (CD62P+), endothelial- (CD31+), and B-cell-derived (CD19+), as well as platelet- and erythrocyte-derived aggregates (CD41+CD235a+). Levels of platelet-derived EVs, expressing both constitutional and activation markers (CD41+CD62P+) decreased in both groups of patients 24 h after surgery. On-pump cardiac procedures led to an increase in T-regulatory cell-derived EVs (CD73+CD39+), suggesting a potential mechanism for immune suppression compared to off-pump surgery. There were numerous correlations between EV levels and cytokine profiles following on-pump surgery, hinting at a close relationship. Leucocyte-derived EVs exhibited positive correlations with each other and with GRO but showed negative correlations with endothelial-derived EVs (CD90+ and CD31+). Additionally, CD73+ EVs demonstrated positive correlations with platelet counts and with erythrocyte-derived CD235a+ EVs. EV changes were significantly greater after on-pump surgery, highlighting a more pronounced response to this type of surgery and emphasizing the role of EVs as regulators of post-surgical inflammation.

## 1. Introduction

While innovations in heart surgery have improved outcomes, the use of cardiopulmonary bypass (CPB) causes a systemic response that may occasionally result in organ failure and worse outcomes [1,2,3,4,5]. Surgical trauma, blood contact with artificial surfaces, and ischemia-reperfusion are all partners in triggering this whole-body inflammation [3,4,5,6,7]. This “inflammatory storm” includes activation of the blood cascade systems such as coagulation, fibrinolysis, and the complement system, diverse blood cells including inflammatory cells, and the release of inflammatory mediators such as cytokines.

There is evidence that off-pump coronary artery bypass grafting (CABG) is associated with a reduced inflammatory response during the procedure compared to on-pump CABG [6,8,9]. For instance, off-pump surgery had less activation of coagulation, fibrinolysis [10], and the complement system [11].

Extracellular vesicles (EVs) are suggested to be able to mediate a series of immune responses and inflammation [12,13,14]. Ischemia-reperfusion induces the release of extracellular vesicles (EVs) in the heart [15]. Moreover, EVs not only amplify local inflammation within the heart but also trigger systemic inflammation [16], causing endothelial dysfunction, coagulation disorders, etc. [17,18]. Furthermore, we found earlier that on-pump surgery released damage-associated molecular patterns (DAMPs) like mitochondrial DNA, which are partly carried in EVs [19]. Thus, EVs may be the driver of inflammatory responses related to various cell activities and cytokine release.

Thus, the majority of research comparing off-pump and on-pump open heart surgery has focused on measuring levels of cytokines [20], and a number of studies focused on the cellular response of the immune system [21], basically only investigating main T- and B-cell subsets and only a few manuscripts are dedicated to the participation of EVs. Meanwhile, understanding the fine-tuning mechanisms of immune response regulation seems to be a key factor in improving the quality of open-heart surgery. The present study aims to comprehensively explore early immune response, including dynamics of T- and B-lymphocyte subsets, various cytokines, and plasma extracellular vesicles, on coronary artery bypass grafting surgery performed in off-pump or on-pump conditions.

## 2. Materials and Methods

### 2.1. Study Design and Patient Characteristics

Thirty-six male patients undergoing coronary artery bypass were enrolled in the study. The research was approved by the local Ethics Committee of Almazov National Medical Research Centre (protocol No. 08122019) and complied with the Helsinki Declaration. All patients included in the study provided informed consent.

All patients scheduled for isolated CABG were considered. The following inclusion criteria were defined: (1) availability of voluntary informed consent signed by the patient; (2) age from 18 to 75 years; and (3) elective isolated CABG (off-pump and on-pump). The exclusion criteria were (1) chronic heart failure (CHF) of functional class IV according to NYHA classification, (2) left ventricular ejection fraction less than 40%, (3) sepsis, and (4) taking immunosuppressive drugs before surgery.

All patients had angina pectoris or had had myocardial infarction. Patient characteristics, including comorbidities, are presented in Table 1.

### 2.2. Anesthesia and CPB

Intraoperative monitoring included a 7-lead electrocardiography with ST segment analysis, invasive blood pressure measurement, pulse oximetry, measurement of nasal and rectal temperatures, as well as measurement of central venous pressure. The control of these indicators was carried out using the system “Philips intellivue MX800” (Philips, Amsterdam, The Netherlands). Anesthesia was induced with a slow infusion of propofol (1.5 mg/kg) in combination with fentanyl (5 mcg/kg) and pipecuronium bromide (0.8 mg/kg). Anesthesia was maintained with a propofol infusion of 6 mg/kg/h. Analgesia was provided with a continuous fentanyl infusion of 5 mcg/kg/h. Mechanical ventilation was performed by using “Draeger Zeus Infinity Empowered” (Draeger, Lübeck, Germany).

CPB was with membrane oxygenators “Maquet” (Rastatt, Germany) and normothermic perfusion (central temperature 36 ± 0.5 °C). Perfusion was with a flow of 2.4–2.6 L/min/m^2^ body surface and a mean arterial pressure of 60–80 mmHg. The quality of CPB was assessed by monitoring blood gases and acid–base balance.

Blood was sampled twice: before surgery and anesthesia and 20–24 h later in the intensive care unit. Venous peripheral blood samples were collected into vacuum test tubes containing K_3_EDTA and were then processed to analyze the relative and absolute numbers of the main T- and B-cell subsets by multicolor flow cytometry. A standard blood test was conducted using a Cell-DYN Ruby Hematology Analyzer (Abbott, Abbot Park, IL, USA). Plasma for further investigation of extracellular vesicles and level of cytokines was obtained as described below no more than four hours after blood collection. T-cell and B-cell immunophenotyping was performed within four hours after sampling.

### 2.3. Extracellular Vesicles

EVs were investigated according to official recommendations from the International Society for Extracellular Vesicles [22,23,24] and as was previously described by our group [25,26,27,28,29]. Plasma was obtained from whole blood collected into vacuum tubes with K_3_EDTA. To avoid residual cell contamination of plasma, each sample was proceeded with sequential centrifugation. In the first step, blood tubes were centrifuged two times successively at 1500× *g* for 10 min at +18 °C and one time at 3000× *g* for 20 min at +4 °C. After each centrifugation step, the supernatants were carefully transferred to new conical tubes. The resulting plasma was aliquoted and stored at −80 °C for further use.

The phenotyping of circulated extracellular vesicles was performed according to the official recommendations from the International Society for Extracellular Vesicles [30,31]. Immunofluorescence staining of the samples was carried out using the following antibodies, mixed in 5 panels and presented in Table 2. All of the panels were performed separately in appropriate tubes. A total of 50 µL of each sample was stained with 0.5 µL of each monoclonal antibody for 25 min at 20 °C in the dark. Single-stained controls were performed to identify whether each fluorochrome in the panel affects measurements of other fluorescent reagents as well as for compensation validation. The isotype controls were as follows: Alexa Fluor 488 Mouse IgG1, κ Isotype Ctrl (BioLegend Inc., San Diego, CA, USA, clone: MOPC-21, cat. 400129, concentration 200 µg/mL), PE Mouse IgG1, κ Isotype Ctrl (BioLegend Inc., San Diego, CA, USA, clone: MOPC-21, cat. 400114, concentration 200 µg/mL), and APC Mouse IgG1, κ Isotype Ctrl (BioLegend Inc., San Diego, CA, USA, clone: MOPC-21, cat. 400122, concentration 200 µg/mL). All isotype controls and single-stained controls were stained and measured in the same conditions and concentrations as the appropriate fluorochrome-marked antibodies.

For each panel of stained samples, the serial dilution procedure was performed [25]. Briefly, 20 µL of stained sample was added to 180 µL of phosphate-buffered saline (PBS) (Biolot, St. Petersburg, Russia), resulting in a 10-fold dilution. This 10-fold dilution was serially diluted with 100 µL of sample and 900 µL of PBS. All diluted samples were phenotyped with at least three dilutions, starting from 10-fold and finishing with 10,000-fold. All of these series were used for calculation of initial plasma EV concentration.

A buffer-only control of PBS (Biolot, St. Petersburg, Russia) and a buffer with reagent controls (0.5 µL of each monoclonal antibody in 50 µL of PBS) were recorded at the same flow cytometer acquisition settings as all other samples, including the triggering threshold, voltages, and flow rate. To allow comparisons between serial diluted stained samples, all these controls were diluted five times with PBS in the same way as the samples. The buffer with reagent control had the same event rate as the buffer-only control. Additionally, unstained controls were measured at the same conditions and dilutions as stained samples. The flow cytometer acquisition settings were unchanged, including the triggering threshold, voltages, and flow rate for all controls and stained samples.

To avoid the effect of coincidence, a working dilution of samples for each staining panel of antibodies was performed. The criteria for optimal dilution were as follows: linear correlation between the dilution factor and event count, absence of differences in fluorescence of the events, and scatter intensity. The achieved optimal dilution for each panel of stained samples was used for processing the results to calculate EV concentration and presenting the number of events per µL (events/µL).

The phenotyping of EVs was conducted on a Cytoflex S (Beckman Coulter, West Sacramento, CA, USA) high-sensitivity flow cytometer as previously described [25,27,29]. The detection was triggered on a 405 nm laser at the threshold of 2000 arbitrary units. Instrument calibration setup was performed using the Cytometry Sub-Micron Particle Size Reference Kit, Molecular probes by Life Technologies), and Megamix-Plus FSC and Megamix-Plus SSC (Biocytex, Marseille, France) containing FITC-labeled reference beads of various diameters from 100 and up to 1000 nm. The sample flow rate was set at 120 µL min^−1^. Stained objects were detected using the side scatter from the violet 405 nm laser and the appropriate use of the fluorochrome-labeled antibodies light pass channel.

Additional controls with detergent were used to prove the membrane structure of investigated objects. Firstly, samples were stained with monoclonal antibodies and phenotyped as described above. Then, equal volumes of either PBS or 2% Triton X100 in PBS were incubated with stained samples at room temperature in the dark for 20 min, followed by the flow cytometry procedure. The loss of at least 90% of events pointed to membrane lysis and proved the EVs nature of studied events.

The results of EV phenotyping were processed using CytExpert v.2.4 (Beckman Coulter, Chaska, MN, USA) and Kaluza 2.1 (Beckman Coulter, Chaska, MN, USA) software. The results of EV concentrations in plasma were presented as appropriate marker positive events per µL. The use of multicolor high-sensitivity flow cytometry allowed us to calculate relative amount of stained with different antibody events. Thus, results from double stained panels were presented as relative number of double-positive and single-positive events, where their sum was taken for 100%.

### 2.4. Cytokines

The levels of plasma cytokines were assessed as previously described [32] using multiplex analysis platform and MILLIPLEX^®^ MAP Human Cytokine/Chemokine/Growth Factor Panel A (HCYTA-60K-PX48, MilliporeSigma, Burlington, MA, USA). Briefly, 25 µL of plasma samples and fluorescently labeled magnetic microsphere beads were added into the appropriate plate wells and incubated with agitation on a plate shaker overnight at +6 °C. The antibodies for detection were added into wells and incubated with agitation for one hour at room temperature in the dark, followed by the incubation with streptavidin-phycoerythrin in the same conditions. The standard samples were prepared according to manufacturer’s recommendations. The calibration curve was created for each detected cytokine. Results were processed on the Luminex MAGPIX^®^ (RUO) Instrument (Luminex, Austin, TX, USA). All the data were generated with xPONENT software (v. 4.2) and analyzed with Milliplex Analyst 5.1 Flex software.

### 2.5. T-Cell Immunophenotyping by Flow Cytometry

Immunophenotyping procedure was performed as previously validated and described by our group [33,34,35,36]. Multicolor flow cytometry was employed to analyze the Th-cell subsets. Whole blood (100 µL) was stained using APC-Alexa Fluor 750-labeled mouse anti-human CD3 (Beckman Coulter, INpolis, IN, USA), Pacific Blue-labeled mouse anti-human CD4 (Beckman Coulter, INpolis, IN, USA), APC-labeled mouse anti-human CD8 (Beckman Coulter, INpolis, IN, USA) and Krome Orange-labeled mouse anti-human CD45 (Beckman Coulter, INpolis, IN, USA) antibodies. Staining procedures were conducted following the manufacturer’s guidelines. Briefly, blood samples were stained with the specified above-mentioned antibodies at room temperature for 15 min, protected from light. Erythrocytes were then lysed using 1 mL of VersaLyse Lysing Solution (Beckman Coulter, Inc., INpolis, IN, USA) and 25 µL IOTest 3 Fixative Solution (Beckman Coulter, Inc., INpolis, IN, USA). Following a two-step wash with PBS containing 2% FCS (Sigma-Aldrich Co., Saint Louis, MO, USA), cells were resuspended in PBS with 2% neutral formalin (Sigma-Aldrich Co., Saint Louis, MO, USA). Flow cytometry analysis was performed on Beckman Coulter Navios EX flow cytometer (Beckman Coulter, West Sacramento, CA, USA) with a minimum of 40,000 CD3+CD4+ T-cells per sample. Main T-cell subsets were identified as T-helper cells (CD45+CD3+CD4+) and T-cytotoxic cells (CD45+CD3+CD8+). Results were presented as absolute and relative amounts of T-cell subsets.

### 2.6. B-Cell Immunophenotyping by Flow Cytometry

The phenotyping of B-cell subsets was performed by flow cytometry after staining 100 µL of whole blood with APC/Cy7-labeled CD19 (BioLegend, Inc., San Diego, CA, USA) and Krome Orange-labeled mouse anti-human CD45 (Beckman Coulter, INpolis, IN, USA) antibodies. A procedure of erythrocyte lysis followed by cell washing was conducted, as described above. A minimum of 5000 CD45+CD19+ B-cells were analyzed per sample.

### 2.7. Statistics

Statistical analysis was performed by using Statistica 7.0 (StatSoft, Oklahoma, OK, USA) and GraphPad Prism 8 (GraphPad Software Inc., San Diego, CA, USA) software. All results were presented as median and interquartile range—Me (25; 75). The differences between groups before and after surgery were analyzed using the Wilcoxon match paired test. Correlation analysis was performed with non-parametric Spearman correlation test. Significances were set at *p* < 0.05.

## 3. Results

### 3.1. Laboratory Characteristics

Laboratory characteristics of the patients are presented in Table 3. In both patient groups, surgery increased the absolute count of leucocytes, neutrophils, and monocytes but decreased lymphocytes and erythrocytes.

### 3.2. Extracellular Vesicles

**Figure 1:**
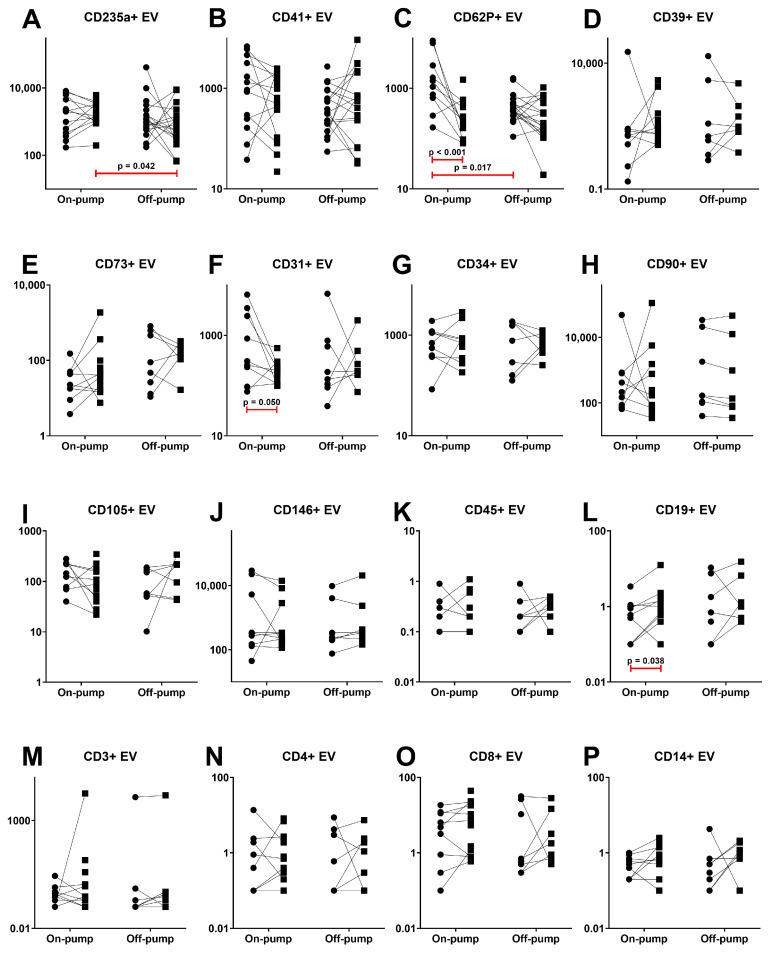
Changes in the levels of different plasma extracellular vesicles after on-pump and off-pump coronary artery bypass surgery. Vesicle type is presented in Table 2. (**A**) CD235a+ EVs. (**B**) CD41+ EVs. (**C**) CD62P+ EVs. (**D**) CD39+ EVs. (**E**) CD73+ EVs. (**F**) CD31+ EVs. (**G**) CD34+ EVs. (**H**) CD90+ EVs. (**I**) CD105+ EVs. (**J**) CD146+ EVs. (**K**) CD45+ EVs. (**L**) CD19+ EVs. (**M**) CD3+ EVs. (**N**) CD4+ EVs. (**O**) CD8+ EVs. (**P**) CD14+ EVs. Circles demonstrate values before surgery, squares—24 h after surgery.

Significant changes in plasma EV concentrations were observed only after on-pump surgery (Figure 1). The levels of CD62P+ EVs and CD31+ EVs decreased, whereas the level of CD19+ EVs increased 24 h after surgery. The level of CD235a+ EVs was lower after off-pump than after on-pump CABG. Noteworthily, the preoperative level of CD62P+ EVs was lower in patients scheduled for off-pump surgery compared to those scheduled for on-pump surgery.

**Figure 2:**
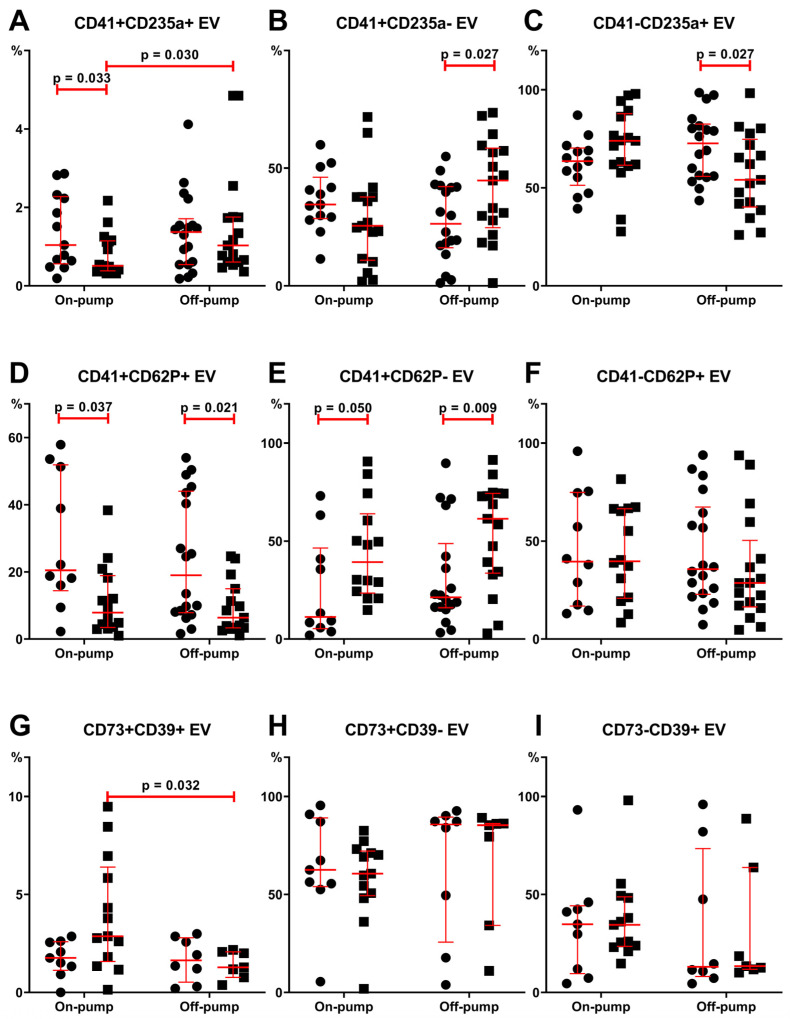
Relative amounts of extracellular vesicles investigated using double-staining protocols of immunophenotyping. Vesicle type is presented in Table 2. (**A**) CD41+CD235a+ EVs. (**B**) CD41+CD235a- EVs. (**C**) CD41-CD235a+ EVs. (**D**) CD41+CD62P+ EVs. (**E**) CD41+CD62P- EVs. (**F**) CD41-CD62P+ EVs. (**G**) CD73+CD39+ EVs. (**H**) CD73+CD39- EVs. (**I**) CD73-CD39+ EVs. Results presented as median and interquartile range before and after on-pump and off-pump CABG. Circles demonstrate values before surgery, squares—24 h after surgery.

Erythrocyte- (CD235a+) and platelet-derived (CD41+) EVs were the most numerous in plasma in both patient groups at both time points (Figure 2). Meanwhile, double-positive events were not higher than 5%. Their level decreased (*p* = 0.033) in patients after on-pump surgery. The level of CD41+CD2235a- EVs increased while CD41-CD235a+ decreased in the off-pump group.

The level of CD41+CD62P+ was the highest among double-positive EVs, with up to 60% of stained platelet-associated markers (CD41 and CD62P) on EVs (Figure 2). The high levels decreased in both groups after surgery. These relative amounts were replaced by CD41+CD62P- EVs since levels of CD41-CD62P+ did not differ in both groups of patients after surgical treatment compared to the preoperative period.

The level of CD73+CD39+ EVs was lower in off-pump patients compared to on-pump patients after surgery.

### 3.3. T- and B-Cells

The proportions of major T-cell subsets did not differ between the two groups of patients before surgery (Table 4). Surgery resulted in a significant decrease in the absolute count of Th cells and cytotoxic T-cells in both groups of patients. Despite the significant elevation of the relative amount of B-cells in both groups of patients, absolute B-cell counts increased only in patients after on-pump surgery.

### 3.4. Cytokines

Levels of plasma cytokines were measured in patients 24 h after CABG (Table 5). Except that IL-15 was higher (*p* = 0.04) after on-pump surgery, there were no significant differences between on- and off-pump surgery.

### 3.5. Correlation Analysis

**Figure 3:**
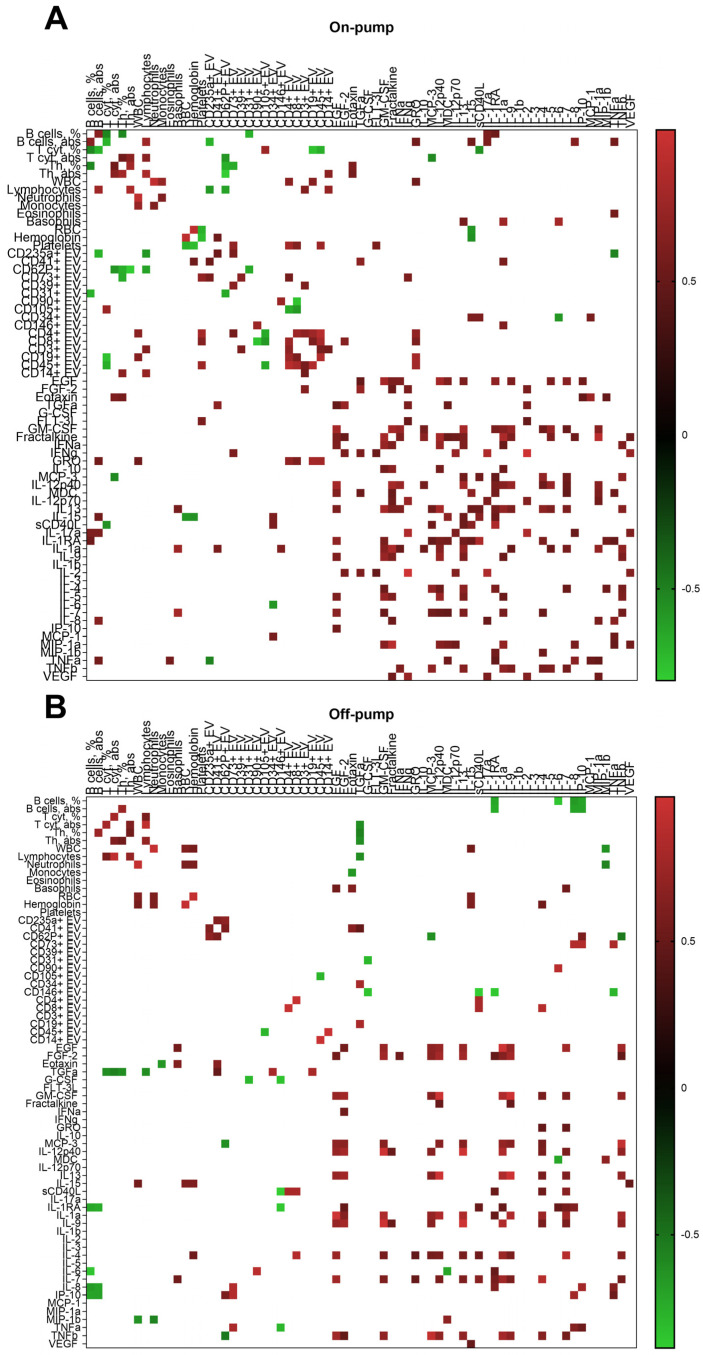
Heatmap of correlations between plasma extracellular vesicles, cytokines, and blood cell parameters in patients after on-pump (**A**) and off-pump (**B**) coronary artery bypass grafting.

Compared to off-pump CABG, on-pump surgery resulted in a greater number of positive correlations, primarily in cytokine levels (Figure 3). Furthermore, platelet-derived CD62P+ EVs and erythrocyte-derived CD235a+ EVs demonstrated negative correlations with T- and B-cells. Additionally, on-pump surgery resulted in numerous cross-correlations between EV levels and limited positive relationships with certain cytokine levels (e.g., GRO, IL-15, sCD40L, IL-1a). These correlations were not observed in patients after off-pump surgery.

## 4. Discussion

Extracellular vesicles act as couriers, delivering bioactive molecules to cells and tissues, potentially triggering a range of effects. These effects can be beneficial, neutral, or harmful, influencing the recipient cell’s behavior. For instance, they can switch cell phenotypes, modulate gene expression, and control biological pathways such as inflammatory cell recruitment, activation of myeloid cells, and cell proliferation [37]. Cargo of cytokines depends on vesicle cell origin and influence on their effects. EVs have been found to carry a diverse array of cytokines, including IL-2, IL-4, IL-10, IL-12, IL-15, IL-16, IL-18, IL-21, IL-22, IL-33, eotaxin, IFN-γ-inducible protein-10, inducible T-cell alpha chemoattractant, M-CSF, MIG, macrophage inflammatory protein-3 α, TGF-β, and TNF-α [38]. Furthermore, EVs originating from T-cells and monocytes specifically contain IL-17, IL-2, IL-12p70, IL-4, C-X-C motif chemokine 11, IL-21, IL-33, IL-22, IFN-γ, TGF-β, and TNF-α [39].

Platelet and erythrocyte-derived EVs (PEVs) are the most numerous populations of particles in blood plasma [40]. PEVs are able to act as modulators of interaction between monocytes and endothelial cells [41]. They activate intracellular cell adhesion molecule-1 on endothelial cells and integrin subunit alpha L (CD11a), integrin subunit alpha M (CD11b), and CD14 on blood monocytes [41]. All of these antigens are crucial in inflammation. P-selectin, a protein found on the surface of PEVs, plays a key role in the aggregation and accumulation. Thus, stimulation of platelets leads to PEV release of transforming growth factor beta (TGF β) that suppresses apoptosis of polymononuclear leucocytes (PMNs) [42,43]. Moreover, PEVs are capable of inducing the adhesion of PMNs on the endothelium. It occurs due to interleukin-1, carried by PEVs and induces endothelial cell adhesiveness to neutrophils [43,44]. Moreover, this extracellular vesicle subset is a source of IL-1, IL-6, and TNF α [45].

Thus, all the above-mentioned features indicate that PEVs are capable of performing strong proinflammatory activity. Nevertheless, there is no doubt now that they also have anti-inflammatory properties that are largely attributed to their ability to suppress the release of cytokines. Lipoxygenase 12 (12-LO) positive PEVs are thought to be involved as mediators in the synthesis of lipoxin A4 (LXA4) by mast cells, which leads to the inhibition of inflammation [46]. Platelet extracellular vesicles decrease the production of the pro-inflammatory proteins TNF-α and IL-10 by macrophages and induce the release of TGF β [47]. These findings suggest that PEVs downmodulate differentiation between monocytes and immature dendritic cells [47]. Interestingly, platelet EVs also demonstrated an inhibitory effect on the adaptive immune system. Regulatory T-cells (Tregs) are suppressed to release IL-17 and interferon gamma (IFN-γ) by PEVs in a P-selectin-dependent manner [48].

All platelets, as well as platelet-derived microvesicles, express CD41 molecules [49], whereas microparticles released from activated cells maintain CD62P and lysosomal-associated membrane protein-1 (LAMP-1) expression [50]. The majority of CD41+ EVs in the plasma lacked expression of CD62P and LAMP-1 [51].

Despite the lack of significant differences in platelet levels after heart surgery in both groups of patients, we observed a decrease in the number of CD62P+ EVs only after on-pump CABG (Figure 1). Interestingly, there were no differences in PEVs defined by expressing the most common platelet marker, CD41. It was even more interesting to study PEVs expressing both markers or, controversially, only one. Thus, a significant decrease in double-positive events was observed after surgery in both off-pump and on-pump groups of patients (Figure 2D). Moreover, single-positive CD41+CD62P- EVs even demonstrated an increase in their plasma levels (Figure 2E) with no differences in CD41-CD62P+ EVs (Figure 2F). Thus, the above-mentioned reduction of CD62P+ EVs after on-pump CABG occurred predominantly due to double-positive events, demonstrating a decrease in microvesicles originating from activated platelets. Investigating the impact of platelet-derived EVs in the pathogenesis of post-surgery complications and pathological conditions, it should be noted that surface densities of phosphatidylserine, CD61, CD62P, and factor X bound per surface area are 2.7-, 8.4-, 4.3-, and 13-fold higher for vesicles than for activated platelets, respectively. Moreover, the PEV surface is 50- to 100-fold more procoagulant than the surface of activated platelets [52].

Our findings suggest that on-pump surgery has a greater impact on the humoral immune response, as reflected in cytokine and EV levels, compared to off-pump surgery. Notably, platelet-derived EVs, while showing negative correlations with T- and B-cells, demonstrated minimal correlation with cytokine levels.

Previously, it was shown that a delayed heparin-induced impairment of platelet aggregation occurred in patients before CPB, suggesting that platelet defects may result from interaction with heparin but not as a result of passage through the bypass circuit [53]. Moreover, the results of quantitative and qualitative analysis of extracellular vesicles may be affected by storage time and anticoagulants used in sample preparation. Heparin is known to be the powerful trigger for in vitro vesiculation by all cell fractions presented in the peripheral blood [54]. CPB has a major impact on coagulation. Patients who undergo on-pump heart surgery with CPB are more likely to experience excessive bleeding compared to those who have surgery off-pump [55]. Various hematological changes take place during CPB because of the passage of blood through the circuit, resulting in activation of coagulation factors, adhesion of blood components, hemodilution, activation, and increased cytokine production by leucocytes, etc. [56]. In our study, levels of potentially highly active platelet-derived CD41+CD62P+ EVs decreased after both off-pump and on-pump surgery, indicating their limited significance in the postoperative period. Moreover, circulating blood through a miniature CPB circuit does not lead to an increase in platelet microvesicle production [57], surgical procedures themselves seem to be a stronger trigger for PMV formation than CPB itself [56]. Meanwhile, our study found that all observed differences in EV concentrations after heart surgery occurred in the on-pump patient group.

Erythrocyte-derived EVs (REVs) play a significant role in a wide range of physiological and pathological activities [58]. REVs contribute to blood clotting by activating clotting factors through a mechanism that depends on coagulation factor XI (FXI). They can initiate and amplify the production of thrombin, a crucial enzyme in the blood clotting process [59]. Transfusions of red blood cells that often take place during open heart surgery and obviously in on-pump procedures can lead to adverse reactions [60,61]. Extracellular vesicles (EVs) released from red blood cells that have been stored for extended periods are strongly associated with the immune and inflammatory reactions that can occur during blood transfusions [28,58]. Some studies have demonstrated that REVs can interact with monocytes, prompting the release of pro-inflammatory cytokines like IL-1, IL-6, and TNF-α, as well as chemokines such as macrophage-derived chemokine (MDC) and macrophage inflammatory protein 1a (MIP-1a), which promote T-cell proliferation and further stimulate T-cells to produce TNF, IL-6, and IL-8 [62].

A commonly used marker for REVs is glycophorin-A (CD235a) [29,49,63], which was also used in our study. Performed CABG revealed a difference in levels of CD235a+ EVs in patients after off-pump and on-pump surgery (Figure 1A). Contrariwise, the concentrations of CD41+CD235a+ EVs after off-pump surgery were higher than those after the on-pump procedure (Figure 2A). The presence of membrane markers of platelet and erythrocyte cell origin simultaneously can be explained by vesicle aggregate formation or molecule transfer performed by microparticles. It is noteworthy that levels of REVs positively correlated with PEVs in both off-pump and on-pump surgeries while showing no correlation with cytokine levels.

Endothelial-derived extracellular vesicles (EEVs) may also be present in peripheral blood and can be defined by high-sensitivity flow-cytometry analysis of the glycoprotein expression E-selectin (CD62E), endoglin (CD105), platelet endothelial cell adhesion molecule 1 (PECAM-1, CD31), vascular cell adhesion molecule 1 (VCAM-1, CD106), vascular endothelial cadherin (VE-cadherin, CD144), melanoma cell adhesion molecule (MCAM, CD146), etc. [17,25,27]. Their activity may be involved in the inflammation process. Thus, the production of EVs by endothelial cells increased in the presence of IL-1α [27], IL-1β [64], IFN γ [64], complement proteins C5b-9 [65], CRP [66], and LPS [64]. The interaction between TNF and EEVs provides a clear example of the inflammatory cycle. TNF stimulates the production of EEVs, which then further enhance the expression of adhesion molecules on endothelial cells, perpetuating the inflammatory process. This cycle underscores the intricate interplay between these factors in inflammation [43]. Thus, TNF triggers an increase in the expression of adhesion molecules, particularly ICAM-1, on both EEVs and the surface of the endothelium. This heightened expression of adhesion molecules leads to a stronger interaction between leukocytes and endothelial cells. Additionally, EEVs demonstrate greater affinity to monocytes compared to neutrophils and lymphocytes [67]. Further, TNF-induced EEVs stimulate endothelial cells to produce proinflammatory cytokines, including interferon gamma-induced protein 10 (IP-10) [68].

Our study revealed only a reduction of CD31+ EVs after on-pump surgery. These results are consistent with a previous study, which demonstrated that in patients with polytrauma and hemorrhagic shock, a significant reduction of CD44+ and CD31+ EVs was observed. Moreover, both EV populations showed a moderate correlation with the transfusion of erythrocyte concentrate [69]. We did not observe predicted correlations between EEV levels and TNF-α or IL-1α and IL-1β. However, we found positive correlations between CD34+ EVs and IL-15, sCD40L, and MCP-1, but only in the context of on-pump surgery. Conversely, off-pump surgery resulted in negative correlations between CD146+ EVs and TNF-α, IL-1RA, and sCD40L.

Limited studies highlight the role of EEVs in adaptive immunity modulation. Their action is realized basically by the activation of lymphocyte proinflammatory pathways through surface antigens [43]. EEVs are capable of inducing the maturation of plasmacytoid dendritic cells (pDCs) [70], which are major producers of type I interferons (IFN-α, IFN-β, IFN-ω) and type III interferons (IFN-λ1, IFN-λ2, IFN-λ3), as well as the inflammatory cytokines IL-6 and TNF α [71,72]. When stimulated with EEVs, pDCs release the inflammatory cytokines IL-6 and IL-8. Additionally, EEVs support the proliferation of CD4+ and CD8+ T-cells [73]. Moreover, the co-incubation of peripheral blood mononuclear cells (PBMC) with EEVs led to an increase in the number of Th1 cells, demonstrating an influence on Th-cell polarization [74]. Correlations between EEVs and main lymphocyte subsets in our study suggest a relationship specific to on-pump surgery, as they were not observed after off-pump procedures.

Surgical trauma causes endothelial and soft tissue injury that leads to the formation of platelet microvesicles [75,76]. Moreover, cardiac surgery trauma contributes to a higher production of microvesicles in addition to the impact of CPB. This is proved by higher concentrations of coagulation markers and microvesicles in pericardial blood than blood in the left ventricle [77,78]. Blood plasma collected after on-pump coronary bypass surgery, but not after off-pump surgery, has been shown to stimulate the production of superoxide radicals within the vascular walls. This effect appears to be linked to the presence of circulating microparticles, likely originating from the endothelium, that persist for at least 24 h following the procedure [79]. However, when investigating endothelial-derived and leukocyte-derived vesicles, we observed an increase in CD19+ EVs and a decrease in CD31+ EVs exclusively after on-pump surgery. All other vesicle subsets, particularly after off-pump procedures, maintained stable concentrations.

Leukocyte-derived extracellular vesicles (LEVs) may originate from monocytes, neutrophils, as well as B- and T-lymphocytes and contribute to hemostasis, angiogenesis, and inflammation [43]. Their activity and effects are significantly related to their cell origin. Early research findings show that leukocyte-derived microvesicles can induce the production of IL-6, MCP-1, and tissue factor (TF) in endothelial cells [80], whereas vesicles released from monocytes and macrophages stimulate airway epithelial cells to produce higher levels of inflammatory mediators, such as IL-8, MCP-1, and ICAM-1 [81]. Monocyte EVs, working in an autocrine and paracrine mode, activate the production of TNF-α and IL-6 by both monocytes and macrophages [82].

The link between inflammation and neutrophil-derived microvesicles is strengthened by mounting clinical and experimental evidence demonstrating that these MVs are produced during sepsis [83,84]. Neutrophil-derived EVs also play a role in inflammation by influencing the production of cytokines by natural killer (NK) cells, reducing the release of IFN γ and TNF α, but enhancing the release of TGF β1 [85].

Activated T-cells are able to produce EVs targeting many cell types. For instance, T-cell-generated EVs stimulate the synthesis of proinflammatory (TNF, IL-1β) as well as anti-inflammatory (secretory interleukin-1 receptor antagonist, sIL-1Ra) cytokines in monocytes [86]. Vesicles produced by T-cells can trigger the release of granules and cytokines (IL-8, oncostatin M) from mast cells (MCs) [87]. Vesicles released by natural CD4+CD25+ regulatory T-cells have been shown to suppress the activity of CD8+ T-cells and antitumor immunity [88]. Regulatory T-cells (Tregs) use their EVs to deliver specific microRNAs, particularly miR-150-5p and miR-142-3p, to dendritic cells (DCs). This transfer alters the immune response in tissues, leading to increased production of the anti-inflammatory cytokine IL-10 and reduced levels of the pro-inflammatory cytokine IL-6 [89]. Extracellular vesicles released from CD19+ B-cells have the ability to weaken the immune response of CD8+ T-cells [90]. Moreover, T-cell-generated EVs decrease NO production, simultaneously increasing ROS production in endothelial cells, thus participating in endothelial dysfunction [43,91].

Among all studied leucocyte-derived EVs, only CD19+ EVs demonstrated a significant increase only after on-pump surgery (Figure 1L). It corresponds to the statement that, similar to infectious diseases, non-infectious inflammatory processes also exhibit elevated levels of T- and B-lymphocyte-derived EVs [43]. In contrast to off-pump surgery, levels of leukocyte-derived EVs (CD4+, CD8+, CD3+, CD19+, CD45+, CD14+) were interconnected after on-pump surgery and positively correlated with GRO levels.

A growing number of molecules involved in immune regulation have been identified on the surface of EVs. In particular, these include the immune checkpoint molecules cytotoxic T lymphocyte antigen 4 (CTLA4) and programmed death ligand 1 (PDL1), the apoptosis-inducing ligand FASL (also known as CD95L), and the ectoenzymes CD39 and CD73, which generate immunosuppressive adenosine from pro-inflammatory and immunoactivating ATP [13,92,93]. Traditionally, CD39 and CD73 are positioned as markers of T-regulatory cells but may also be expressed on B-lymphocytes, NK cells, conventional CD4+ T-helper, and CD8+ T-cytotoxic cells [93].

Treg cell EVs exert their immunosuppressive effects through various mechanisms, including the surface expression of CD39 and CD73, which is crucial for adenosine production [94,95]. Similar to cells, it was hypothesized that EVs also require co-expression of CD39 and CD73 on their surface to effectively exert immunoregulatory properties [94]. The proportion of CD73+CD39+ EVs was below 5% in both patient groups, both before and after surgery (Figure 2G), which is consistent with previous findings [94]. In our study, the level of double-positive EVs was higher after on-pump surgery compared to off-pump surgery, demonstrating improved immunoregulatory properties.

## 5. Conclusions

Off-pump cardiopulmonary bypass induced a less pronounced immune response compared to on-pump surgery. EV changes were significantly greater, primarily following on-pump surgery. Our investigation of double-positive EVs revealed distinct patterns: platelet-derived CD41+CD62P+ EVs exhibited similar changes in both surgical groups, while platelet- and erythrocyte-derived aggregates (CD41+CD235a+) significantly decreased only after on-pump surgery. Interestingly, T-regulatory cell-derived CD39+CD73+ EVs, known for their immunosuppressive functions, demonstrated contrasting dynamics, increasing after on-pump procedures. Moreover, on-pump surgery led to greater changes in circulating EVs, particularly platelet- (CD62P+), endothelial- (CD31+), and B-cell-derived (CD19+). There were numerous correlations between EV levels and cytokine profiles following on-pump surgery, hinting at a close relationship. Leucocyte-derived EVs exhibited positive correlations with each other and with GRO but showed negative correlations with endothelial-derived EVs (CD90+ and CD31+). Additionally, CD73+ EVs demonstrated positive correlations with platelet counts and with erythrocyte-derived CD235a+ EVs. EV changes were significantly greater after on-pump surgery, highlighting a more pronounced response to this type of surgery and emphasizing the role of EVs as regulators of post-surgical inflammation. This study revealed patterns in the early postoperative immune response, highlighting the crucial role of extracellular vesicles in its regulation. Understanding the fine-tuning mechanisms of this immune response holds significant promise for improving open-heart surgery outcomes.

## Figures and Tables

**Table 1:** Patient characteristics.

Parameters	On-Pump Surgery, *n* = 18	Off-Pump Surgery, *n* = 18
Median age	64 (59; 69)	68 (65; 75)
Median number of shunts (min–max)	3 (2–5)	3 (1–4)
Diabetes mellitus	3 (16%)	6 (33%)
Dyslipidemia	8 (44%)	9 (50%)
Obesity	5 (28%)	2 (11%)
Chronic gastritis, remission	15 (83%)	14 (78%)
Arterial hypertension	18 (100%)	18 (100%)
Myocardial infarction in anamnesis	11 (61.1%)	12 (66.7%)
Left ventricular ejection fraction, (%)	63 (58; 71.5)	60 (57; 68)

**Table 2:** Panels of monoclonal antibodies for immunophenotyping of EVs.

Antibody	Manufacture	Receptor Specification	Cell Origin
Panel 1			
CD235a PE	BioLegend, San Diego, CA, USA	glycophorin A	Erythroid precursors and erythrocytes
CD41 AF488	BioLegend, San Diego, CA, USA	α subunit of the gpIIb/IIIa (CD41/CD61) complex	Platelets and megakaryocytes
Panel 2			
CD41 AF488	BioLegend, San Diego, CA, USA	α subunit of the gpIIb/IIIa (CD41/CD61) complex	Platelets and megakaryocytes
CD62P PE	BD Pharmingen, Franklin Lakes, NJ, USA	Type I transmembrane glycoprotein; P-selectin; platelet activation-dependent granule membrane protein (PADGEM); GMP-140	Activated platelets, megakaryocytes, and endothelial cells
Panel 3			
CD39 FITC	BioLegend, San Diego, CA, USA	Ecto nucleotidase that can hydrolyze both nucleoside triphosphates and diphosphates	Activated lymphocytes, regulatory T-cells, a subset of T-cells and B-cells, and dendritic cells
CD73 PE	BD Pharmingen, Franklin Lakes, NJ, USA	Ecto-5′-nucleotidase that converts adenosine monophosphate (AMP) to adenosine	Regulatory T-cells, subsets of T- and B-cells, mesenchymal stem cells, follicular dendritic cells, endothelial cells, and epithelial cells
Panel 4			
CD31 FITC	BioLegend, San Diego, CA, USA	Platelet endothelial cell adhesion molecule-1 (PECAM-1)	Monocytes, platelets, granulocytes, endothelial cells and lymphocyte subsets
CD34 PE/Dazzle	BioLegend, San Diego, CA, USA	Type I monomeric sialomucin-like glycophosphoprotein	hematopoietic stem/progenitor cells, bone marrow stromal cells, capillary endothelial cells, embryonic fibroblasts, some nervous tissue
CD90 PE/Cy7	BioLegend, San Diego, CA, USA	GPI-anchored protein, Thy-1	Neuronal cells, a subset of CD34+ cells, fibroblasts, activated endothelial cells
CD105 APC	BioLegend, San Diego, CA, USA	Type I integral membrane homodimer protein, a component of the TGF-β receptor system	vascular endothelial cells, activated monocytes, tissue macrophages, activated endothelium
CD146 PE	BioLegend, San Diego, CA, USA	Integral transmembrane glycoprotein that belongs to the immunoglobulin superfamily	Epithelial cells, endothelial cells, fibroblasts, activated T-cells, multipotent mesenchymal stromal cells, and activated keratinocytes
Panel 5			
CD45 KrO	Beckman Coulter, West Sacramento, CA, USA	Single chain type I membrane glycoprotein, leukocyte common antigen (LCA)	All hematopoietic cells, except erythrocytes and platelets
CD3 AlexaFluor750	Beckman Coulter, West Sacramento, CA, USA	CD3/T-cell receptor (TCR) complex, member of the immunoglobulin superfamily	All mature T-cells, NKT cells, and some thymocytes
CD4 PE	Beckman Coulter, West Sacramento, CA, USA	Single-chain type I transmembrane glycoprotein, member of the Ig superfamily	Most thymocytes, a subset of T-cells, and monocytes/macrophages, T-helper cells marker
CD8 PC5.5	Beckman Coulter, West Sacramento, CA, USA	Type I glycoprotein, a member of the immunoglobulin superfamily	Majority of thymocytes, a subset of peripheral blood T-cells, and NK cells, T-cytotoxic cells marker
CD19 FITC	BioLegend, San Diego, CA, USA	Type I transmembrane glycoprotein, a member of the immunoglobulin superfamily	B-cells, follicular dendritic cells
CD14 APC	Beckman Coulter, West Sacramento, CA, USA	Glycosylphosphatidylinositol (GPI)-linked membrane glycoprotein, LPS receptor	High levels on monocytes and macrophages, and at lower levels on granulocytes

**Table 3:** Routine laboratory test results in patients undergoing on- and off-pump coronary artery bypass grafting.

Parameter, Units	On-Pump Surgery		Off-Pump Surgery		Significance
	Before	After	Before	After	
RBC, ×10^12^	4.17 (3.89; 4.28)	3.42 (3.19; 3.93)	4.26 (4.11; 4.46)	3.55 (3.11; 4.18)	*p*_1,2_ = 0.05 *p*_3,4_ < 0.01
Hemoglobin, g/L	125 (124; 128)	106 (99; 117)	134 (119; 135)	112 (92; 123)	*p*_1,2_ = 0.05 *p*_3,4_ < 0.01
Platelets, ×10^9^	151 (134; 203)	125 (114; 161)	177 (149; 219)	137 (115; 207)	*p*_1,2_ = 0.03
WBC, ×10^9^	5.56 (5.18; 6.68)	13.97 (13.10; 15.28)	6.13 (5.78; 7.51)	12.20 (9.20; 14.30)	*p*_1,2_ = 0.01 *p*_3,4_ < 0.01
Lymphocytes, ×10^9^	1.76 (1.338; 2.03)	1.38 (0.97; 1.96)	1.81 (1.41; 2.79)	1.05 (0.71; 1.74)	*p*_3,4_ < 0.01
Neutrophils, ×10^9^	3.24 (2.55; 3.75)	11.12 (10.61; 12.10)	3.37 (2.35; 3.99)	9.70 (8.03:10.70)	*p*_1,2_ = 0.01 *p*_3,4_ < 0.01
Monocytes, ×10^9^	0.50 (0.44; 0.65)	1.00 (0.65; 1.35)	0.56 (0.49; 0.69)	0.80 (0.36; 1.07)	*p*_1,2_ = 0.01
Eosinophils, ×10^9^	0.09 (0.07; 0.11)	0 (0; 0)	0.07 (0.04; 0.14)	0 (0; 0.01)	*p*_1,2_ = 0.01 *p*_3,4_ < 0.01
Basophils, ×10^9^	0.05 (0.01; 0.09)	0.02 (0.01; 0.09)	0.06 (0.03; 0.09)	0.04 (0.03; 0.05)	

**Table 4:** The relative and absolute count of T-helper, T-cytotoxic, and B-cell subsets before and after coronary artery bypass grafting.

T-Cell Subsets		On-Pump		Off-Pump		Significance
		Before Surgery	After Surgery	Before Surgery	After Surgery	
Th	%	45.1 (37.2; 51.3)	43.7 (34.2; 47.5)	43.8 (38.6; 52.4)	37.4 (30.4; 43.2)	*p*_3,4_ = 0.01
	#	741.4 (707.8; 784.6)	470.0 (331.7; 830.4)	809.8 (536.9; 991.1)	476.3 (345.0; 723.5)	*p*_1,2_ = 0.02 *p*_3,4_ = 0.04
Tcyt	%	21.3 (16.8; 36.0)	21.9 (15.4; 29.1)	23.8 (13.8; 27.1)	20.5 (14.9; 27.9)	*p*_1,2_ = 0.01
	#	417.2 (241.2; 677.9)	301.9 (258.3; 336.0)	415.9 (217.8; 537.9)	221.0 (106.8; 452.0)	*p*_1,2_ = 0.04 *p*_3,4_ < 0.01
B-cells	%	8.7 (5.9; 9.4)	21.0 (12.6; 27.1)	9.5 (6.8; 11.4)	19.5 (7.5; 20.3)	*p*_1,2_ = 0.01 *p*_3,4_ = 0.04
	#	130.5 (99.4; 183.7)	236.5 (119.4; 370.4)	155.7 (81.5; 300.8)	209.1 (107.8; 353.1)	*p*_1,2_ = 0.01

**Table 5:** Plasma levels of cytokines in patients after coronary artery bypass grafting.

Cytokine, pg/mL	On-Pump	Off-Pump
sCD40L	111.1 (89.5; 128.5)	111.1 (89.5; 126.3)
EGF	0 (0; 0)	0 (0; 0)
Eotaxin	168.1 (158.1; 200.7)	178.6 (145.2; 184.5)
FGF-2	120.5 (0; 138.9)	0 (0; 0)
FLT-3Ligand	0 (0; 28.5)	0 (0; 0)
Fractalkine/CX3CL1	0 (0; 0)	0 (0; 0)
G-CSF	209 (199; 233)	199 (141; 238)
GM-CSF	0 (0; 0)	0 (0; 0)
GROa/CXCL1	661 (344; 949)	586 (395; 950)
IFN α2	0 (0; 0)	0 (0; 0)
IFN γ	0 (0; 0)	0 (0; 0)
IL-1α	0 (0; 7.0)	0 (0; 0)
IL-1β	0 (0; 0)	0 (0;0)
IL-1RA	10.4 (7.1; 10.4)	7.1 (0; 14.7)
IL-2	0 (0; 0)	0 (0; 0)
IL-3	0 (0; 0)	0 (0; 0)
IL-4	119.4 (0; 157.2)	0 (0; 146.0)
IL-5	0 (0; 0)	0 (0; 0)
IL-6	136.1 (129.6; 160.6)	151.7 (141.2; 180.6)
IL-7	0 (0; 7.5)	0 (0; 6.7)
IL-8/CXCL8	94.6 (87.9; 102.8)	94.3 (87.5; 101.5)
IL-9	0 (0; 0)	0 (0; 0)
IL-10	55.9 (33.5; 62.5)	59.6 (33.5; 73.6)
IL-12 p40	0 (0; 0)	0 (0; 0)
IL-12 p70	0 (0; 0)	0 (0; 0)
IL-13	0 (0; 0)	0 (0; 0)
IL-15	7.1 (4.1; 10.4) *	4.9 (2.6; 6.8) *
IL-17A	0 (0; 0)	0 (0; 0)
IP-10/CXCL10	327 (241; 508)	368 (234; 443)
MCP-1/CCL2	273 (234; 462)	297 (271; 357)
MCP-3/CCL7	93.3 (0; 97.3)	0 (0; 105.8)
MDC/CCL22	462 (398; 577)	382 (344; 470)
MIP-1a/CCL3	0 (0; 0)	0 (0; 0)
MIP-1b/CCL4	27.8 (18.5; 30.8)	25.2 (19.2; 39.4)
TGF α	0 (0; 0)	0 (0; 0)
TNF α	19.9 (15.9; 21.3)	14.1 (11.6; 20.9)
TNF β	0 (0; 0)	0 (0; 3.2)
VEGF-A	0 (0; 0)	0 (0; 0)

Note: * *p* = 0.04.

## Data Availability

The original contributions presented in the study are included in the article, and further inquiries can be directed to the corresponding author.
